# Atypical Antipsychotics and Metabolic Syndrome: From Molecular Mechanisms to Clinical Differences

**DOI:** 10.3390/ph14030238

**Published:** 2021-03-08

**Authors:** Marco Carli, Shivakumar Kolachalam, Biancamaria Longoni, Anna Pintaudi, Marco Baldini, Stefano Aringhieri, Irene Fasciani, Paolo Annibale, Roberto Maggio, Marco Scarselli

**Affiliations:** 1 Department of Translational Research and New Technologies in Medicine and Surgery, University of Pisa, 56126 Pisa, Italy; k.shivakumar@alumni.sssup.it (S.K.); biancamaria.longoni@med.unipi.it (B.L.); anna.pintaudi@hotmail.com (A.P.); marcobuniversita@gmail.com (M.B.); stefano.aringhieri@gmail.com (S.A.); 2 Department of Biotechnological and Applied Clinical Sciences, University of L’Aquila, 67100 L’Aquila, Italy; irene.fasciani@hotmail.it (I.F.); roberto.maggio@univaq.it (R.M.); 3 Max Delbrück Center for Molecular Medicine, 13125 Berlin, Germany; paolo.annibale@mdc-berlin.de

**Keywords:** atypical antipsychotics (AAPs), G protein-coupled receptors (GPCRs), metabolic syndrome (MetS), weight gain, type 2 diabetes, dyslipidemia, clozapine, olanzapine

## Abstract

Atypical antipsychotics (AAPs) are commonly prescribed medications to treat schizophrenia, bipolar disorders and other psychotic disorders. However, they might cause metabolic syndrome (MetS) in terms of weight gain, dyslipidemia, type 2 diabetes (T2D), and high blood pressure, which are responsible for reduced life expectancy and poor adherence. Importantly, there is clear evidence that early metabolic disturbances can precede weight gain, even if the latter still remains the hallmark of AAPs use. In fact, AAPs interfere profoundly with glucose and lipid homeostasis acting mostly on hypothalamus, liver, pancreatic β-cells, adipose tissue, and skeletal muscle. Their actions on hypothalamic centers via dopamine, serotonin, acetylcholine, and histamine receptors affect neuropeptides and 5′AMP-activated protein kinase (AMPK) activity, thus producing a supraphysiological sympathetic outflow augmenting levels of glucagon and hepatic glucose production. In addition, altered insulin secretion, dyslipidemia, fat deposition in the liver and adipose tissues, and insulin resistance become aggravating factors for MetS. In clinical practice, among AAPs, olanzapine and clozapine are associated with the highest risk of MetS, whereas quetiapine, risperidone, asenapine and amisulpride cause moderate alterations. The new AAPs such as ziprasidone, lurasidone and the partial agonist aripiprazole seem more tolerable on the metabolic profile. However, these aspects must be considered together with the differences among AAPs in terms of their efficacy, where clozapine still remains the most effective. Intriguingly, there seems to be a correlation between AAP’s higher clinical efficacy and increase risk of metabolic alterations. Finally, a multidisciplinary approach combining psychoeducation and therapeutic drug monitoring (TDM) is proposed as a first-line strategy to avoid the MetS. In addition, pharmacological treatments are discussed as well.

## 1. Introduction

Antipsychotic (AP) medications are associated with relevant metabolic side effects, where the so-called metabolic syndrome (MetS) can be responsible for poor adherence, sub-optimal and discontinuation drug use, resulting in relapse and poor clinical outcome. In fact, about 20–50% of patients suffering from schizophrenia or other psychotic disorders, in the long-term, may suspend drug usage with a detrimental effect for their prognosis [1].

In addition, MetS, which has an occurrence of about 40% in chronic schizophrenic patients, has a relevant impact on their general health conditions [1,2]. MetS is defined by the presence of metabolic abnormalities, such as large waist circumference, dyslipidemia, fasting hyperglycemia and elevated blood pressure. Schizophrenic patients have higher morbidity and mortality compared to the general population, where cardiovascular problems are the main cause of this decease and the estimated life expectancy drops down by 10–20 years [3]. However, recent data have confirmed that long term use of atypical antipsychotics (AAPs) in the schizophrenic population is associated with decreased hospitalization and mortality compared to untreated patients, especially with the use of clozapine [4,5].

The relationship between MetS and schizophrenia is complex and multifactorial, where the use of APs has a major role. Besides drugs side effects, other factors such as unhealthy lifestyle, reduced physical activity, smoking, improper diet and genetic predisposition also contribute to metabolic disturbances [6].

APs are generally divided into typical antipsychotics (TAPs) or first-generation APs and atypical antipsychotics (AAPs) or second-generation APs, based on the evidence that AAPs rarely induce motor side effects. However, this distinction has been questioned by many, so a new classification among AAPs was recently proposed by introducing the concept of spectrum of atypia that begins with risperidone (the least atypical) and ends with clozapine (the most atypical), which is still the gold standard for the treatment-resistant schizophrenia [7].

Unfortunately, AAPs higher efficacy on cognition and negative symptoms of schizophrenia is outweighed by the frequent occurrence of MetS and weight gain, thereby limiting their use in clinical practice and compelling clinicians for constant monitoring of patient conditions. It is quite surprising that these relevant side effects, in particular for AAPs, have been underestimated in the past, but now finally clinicians are addressing these concerns while treating psychotic disorders.

Different meta-analysis have shown that the use of AAPs is associated with the highest risk of MetS compared with TAPs, with clozapine and olanzapine being the worse [8]. Importantly, type 2 diabetes (T2D) is not strictly correlated with adiposity. In fact, 25% of patients develop hyperglycemia without gaining weight and this metabolic alteration can happen in the early period of the treatments and precede weight gain [9]. On the other hand, it is important to underline, in the long-term, how weight gain induced by AAPs undoubtedly represents an aggravating factor in the whole metabolic regulation.

The molecular and cellular mechanisms responsible for these metabolic changes are complex and involve practically all the organs relevant for metabolism, including the central and peripheral nervous system.

Indeed, AAPs are drugs targeting many receptors and other proteins, and significantly affect the activities of many hormones and neuromodulators. Different affinities of AAPs on dopamine, serotonin, muscarinic, adrenergic, histamine receptors and other molecular targets (e.g., AMPK) are responsible of their diverse clinical profiles. These receptor targets are well expressed in the hypothalamic centers, pancreas, liver, adipose tissue and skeletal muscle where they modulate glucose and lipid homeostasis in the whole body [10].

With these premises, this review aims to analyze the occurrence of MetS during the use of AAPs by trying to understand the responsible mechanisms in order to identify new potential targets to avoid and/or reduce this undesired effect.

A multidisciplinary approach combining psychoeducation and therapeutic drug monitoring (TDM) is proposed as a first-line strategy. In addition, pharmacological treatments are discussed as well.

## 2. A New Classification for AAPs: The Spectrum of Atypia

Since the discovery of clozapine in the 70’s, the concept of AAPs or second-generation APs was introduced, referring to a new class of medications that were better tolerable, especially in terms of motor-related side effects. However, in the following years, it emerged that the benefits of this new class of drugs were somehow overshadowed due to induced metabolic side effects such as obesity and diabetes. In addition, clinical evidence underlined the diversity within the class of AAPs in terms of efficacy and motor and endocrine-related side effects, highlighting how each AAP was unique and therefore the differences among TAPs and AAPs were questionable [11].

**Figure 1:**
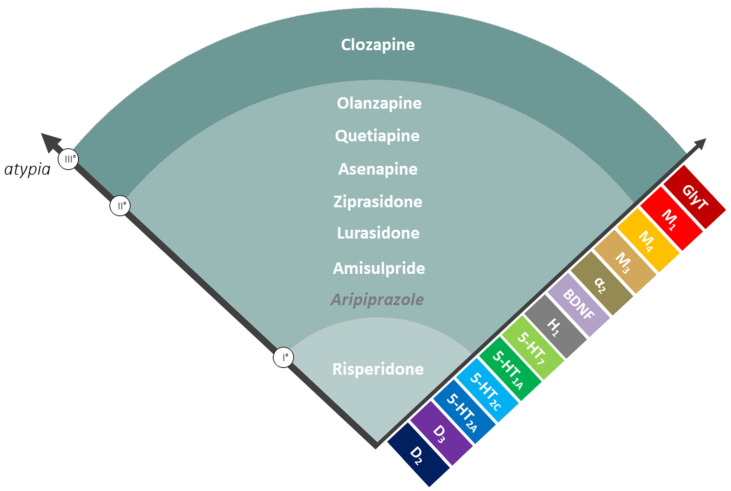
The concept of spectrum of atypia was recently introduced to classify atypical antipsychotics (AAPs). They can be divided in three categories, where risperidone is least atypical (Level I) and clozapine is most atypical (Level III), while all others fall within these two extremes of the spectrum (Level II). The molecular targets shown on the right add up, beginning with the D2 and 5-HT2A,C receptors that are common targets for all AAPs, extending to additional mechanisms such as M1 positive allosterism and GlyT (glycine transporter) activity that seem specific to clozapine. Other targets, such as H1 and α2 receptors and BDNF, are relevant to both Level II and III of atypia [7].

To reconcile the concept of atypicality and diversity among the class of AAPs, a new classification for AAPs was recently proposed by introducing the notion of spectrum of atypia, that ranges from risperidone, the least atypical, to clozapine, the most atypical, while all the other AAPs fall within the extremes of this spectrum (Figure 1). Notably, risperidone and amisulpride can lose their atypicality at higher doses [7]. With this clarification, the concept of atypia is still intact in its essence referring to a category of APs, which demonstrate reduced motor problems, reduced hyperprolactinemia, and reduced worsening of apathy and anhedonia along with a better improvement of negative and cognitive symptoms of schizophrenia. The most effective AAP is still clozapine, which has unique clinical properties to treat drug-refractory schizophrenia, psychoses associated with Parkinson’s disease (PD) and tardive dyskinesia. Unfortunately, these advantages have to be balanced with the increased risk of the AAPs-induced MetS, which is seen less with TAPs [12].

In fact, in clinical practice, many doctors make decisions for a tailored therapy according to the patient’s characteristics, drug efficacy and risks of drug-related side effects. As a consequence, the choice among the different APs is often made by trying to avoid the risk of motor-related side effects or metabolic abnormalities.

Regarding the mechanism of action, AAPs are weak D_2_ receptor blockers and they act beyond D_2_ antagonism, involving other receptor targets (e.g., serotonin (5-HT) receptors) [13,14,15,16]. The ratio of 5-HT_2A_/D_2_ and 5-HT_2C_/D_2_ receptor affinity together with a rapid dissociation constant (Koff) from the D_2_ receptor are two important factors that distinguish AAPs in terms of efficacy and side effects [17,18]. In addition, other molecular targets characterize the receptor profile of the ideal AAP, and among them, 5-HT_1_ partial agonism, H_1_ antagonism, α_2_ antagonism, muscarinic antagonism and positive allosterism, brain-derived neurotrophic factor (BDNF) production, and glycine transporter (GlyT) blocking are relevant for AAP’s action. Clozapine has a unique receptor profile on these molecular targets and therefore it is still considered the gold standard, especially for treatment-resistant schizophrenia. Recently, new concepts such as biased agonism and receptor dimerization have been introduced to explain the differences among different APs [19,20,21]. It has been found that some AAPs have biased signaling activities at D_2_ and 5-HT_2A_ receptors, allowing them to preferentially block or activate specific receptor-mediated intracellular signaling pathways. For instance, clozapine has been shown to act as a biased agonist in vivo and in vitro at 5-HT_2A_ receptor and to activate ERK and Akt kinases, and this can be another factor determining clozapine singularity [19,22].

## 3. Role of GPCRs in the Metabolism of Peripheral Tissues

Glucose and lipid metabolism relies on the coordination between several organs, including pancreatic β-cells, liver, adipose tissue, and skeletal muscle. In addition, the central nervous system (CNS), through the hypothalamic centers via the autonomic nervous system and the neuroendocrine system, constantly regulates their activity.

Besides insulin and glucagon, the main protagonists for energetic homeostasis, other modulators determine a fine regulation of the metabolism by mostly acting on the G protein-couled receptors (GPCRs) that are targeted by AAPs.

For this reason, we briefly summarize the relevance of several GPCRs involved in the metabolism of peripheral tissues, such as glucagon (GCG) receptors, glucagon-like peptide-1 (GLP-1) receptors, gastric inhibitory polypeptide (GIP) receptors, muscarinic receptors, free fatty acid (FFA) receptors, adrenergic receptors, dopamine receptors, serotonin receptors, and ghrelin receptors.

### 3.1. GPCRs and Pancreatic β-Cells

**Figure 2:**
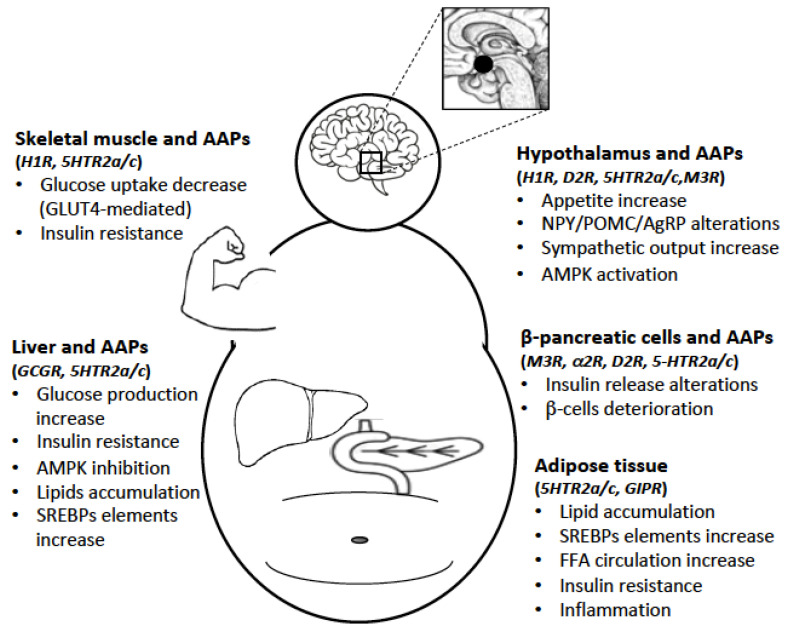
The AAPs-induced metabolic syndrome (MetS) is the consequence of a broad activity of these drugs on the central nervous system (CNS) and peripheral organs. In the CNS, the most important target is the hypothalamus, while in the periphery, the liver, pancreatic β-cells, adipose tissue and skeletal muscle are implicated in AAPs-induced MetS. By interfering with the activity of different GPCRs expressed in these regions, AAPs significantly alter glucose and lipid homeostasis.

Many GPCRs expressed in the pancreatic β-cells are involved in the regulation of pancreatic islet’s function, and therefore they are potential targets responsible for AAPs-induced side effects (Figure 2).

The endogenous agonists for GLP-1 receptors are the incretin hormones released by the intestinal system post-meal ingestion, which enhance the glucose-stimulated insulin secretion (GSIS) by β-cells [23]. The GLP-1 receptor increases GSIS by activating the Gs protein and consequently increasing cAMP. In addition, GLP-1 activates Akt and increases the expression of PDX1 thus causing a cytoprotective and proliferative effect. The GIP receptor has similar characteristics to GLP-1 and is coupled to Gs protein, which induces an increase in cAMP and insulin secretion [24].

On β-cells physiology, the Gs-coupled glucagon receptor is important for regulating insulin secretion, and it seems to have a permissive effect, which guarantees a minimum concentration of intracellular cAMP necessary for the β-cells activity. Indeed, in pancreatic islets isolated from glucagon receptor knockout (KO) Gcgr(−/−) mice, the insulin responses to glucose and several insulin secretagogues were found to be significantly reduced when compared with wild-type mice [24,25].

The most expressed muscarinic receptor subtype is the M_3_, coupled to Gq protein. Studies in mice have clearly shown that it is involved in the correct maintenance of glucose homeostasis [26]. Several studies have shown that an increase in parasympathetic activity can increase insulin secretion during the first minutes of food ingestion [27].

Similar to M_3_, the FFA receptor GPR40, highly expressed in β-cells, is coupled to Gq protein and it enhances insulin secretion by increasing intracellular Ca^2+^ concentration. It may also effect cell vitality through the activation of the intracellular signaling pathways of ERK, PI3K and Akt [28]. The other FFA receptor GPR119 expressed in β-cells is associated with a Gs protein, and it increases insulin secretion by rising the intracellular levels of cAMP [24].

Regarding adrenergic receptors, the two catecholamines, noradrenaline and adrenaline, inhibit insulin secretion via α_2_ receptors coupled to Gi protein; however, the presence of β_2_-adrenergic receptor in the β-cell might indicate its possible role in insulin secretion under certain conditions. In animal models, selective KO studies have shown that the α_2A_ and α_2C_ subtypes mediate the inhibition of insulin secretion by catecholamines [24].

While the role of sympathetic and parasympathetic systems has been clearly demonstrated in β-cell physiology, an intriguing activity of peripheral dopamine and serotonin on β-cells has also emerged over the last two decades [29].

In murine species, the expression of the five dopamine receptors in islets and β-cell cultures, such as INS-1 and MIN6, has been determined using RT-PCR [30]. In particular, the most expressed subtypes are D_2_ (long and short) and D_3_. A subtle inhibitory paracrine and autocrine function on β-cells has been postulated for local dopamine sourced from dietary precursors (e.g., L-DOPA) and/or noradrenergic fibers [31,32,33]. In fact, rodent and human islets express enzymes such as tyrosine hydroxylase (TH), L-aromatic amino acid decarboxylase (AADC), vesicular monoamine transporter 2 (VMAT2) and dopamine active transporter (DAT) to produce and store dopamine [31].

Recently, it was proved that both the D_2_ and D_3_ receptors are relevant for inhibiting GSIS that occurs after the L-DOPA treatment. While the D_2/3_ agonist quinpirole induced a dose-dependent inhibition of GSIS, the D_2/3_ antagonist raclopride was able to block the inhibitory action of L-DOPA and thus restore the normal levels of insulin [34].

In relation to serotonin receptors, different subtypes have been detected in β-cells across different species. However, among them, 5-HT_1A/1D/1F_, 5-HT_2A_ and 5-HT_3_ seem most relevant as their expression may change in response to the metabolic condition [35].

Similar to dopamine, serotonin released mostly by intrapancreatic nerves originated from the enteric nervous system and/or co-released with insulin by β-cells can influence insulin secretion [36,37]. In fact, β-cells express tryptophan hydroxylase (TPH) [38] and AADC, the two enzymes required for serotonin synthesis. Additionally, similar to dopamine, serotonin can be stored in insulin vesicles via VMAT2 and it can be reuptaken by serotonin transporter (SERT) [37].

Intriguingly, the role of serotonin in insulin secretion is still not clear because both in vitro and in vivo studies have found different and contradicting results. It seems that the administration of serotonin can either reduce or increase GSIS based on expression of different serotonin receptor subtypes [37]. In β-cell cultures, such as MIN6 and INS-1, the 5-HT_2_ selective agonists reduced GSIS whereas in vivo they produced an opposite effect [39].

### 3.2. GPCRs and Liver

The liver is the main source of glucose production, and glucagon and insulin constantly regulate its activity as a consequence of plasma nutrients levels. Besides, other GPCRs also influence its metabolic activity (Figure 2).

Glucagon receptors cause an increased expression of cAMP, PKA, and CREB, with consequent activation of gluconeogenesis, glycogenolysis and inhibition of glycolysis. In addition, the cAMP-PKA-CREB pathway also provides a strong stimulus for the turnover of amino acids in the liver by activating enzymes of the urea cycle [40]. In the liver, the presence of incretin receptors such as GLP-1 or GIP do not seem significant, whereas ghrelin receptors are capable of influencing hepatic lipid metabolism by increasing lipogenesis [41].

The sympathetic and parasympathetic activity on liver metabolism is complex and still controversial. The β_2_ receptors expressed in hepatocytes influence carbohydrate and lipid metabolism, and these receptors seem to prevent the accumulation of lipids in mice receiving fat diet, therefore they are a plausible target for treating fatty liver disease [42]. In fact, genetic deletion of β_2_ adrenergic receptor aggravates hepatic lipid accumulation and it has been hypothesized that the lack of β_2_ alters CREB-mediated regulation of peroxisome proliferator-activated receptor PPARγ activity in the liver [43].

Regarding muscarinic receptors, acetylcholine alters the hepatic energy metabolism by stimulating glycogen synthesis, apparently through the Gq-coupled M_3_ receptor [44]. Similar to β_2_, the M_3_ can reduce hepatic lipid accumulation via AMPK activation [45]. Recently, it was suggested that muscarinic receptors are not relevant for maintaining glucose homeostasis as, surprisingly no significant metabolic differences were found in M_3_ mutant mice compared to control group, and in addition, the expression of key genes for regulation of liver metabolism remained unaltered [46]. On this topic, it was proposed that other GPCRs coupled to Gq protein, such as vasopressin V1b receptor, can be relevant to regulate hepatic glucose production.

Another GPCR family expressed in hepatocytes are serotonin receptors, namely 5-HT_2A/2B_ and 5-HT_3_. Activation of the 5-HT_2A/2B_ promotes gluconeogenesis and inhibits the absorption of glucose, thereby increasing blood sugar during fasting periods [47,48]. Serotonin promotes a similar activity through 5-HT_3_. In addition, serotonin regulates the hepatic accumulation of triglycerides via mTOR pathway [49].

### 3.3. GPCRs and Adipose Tissues

The presence of GIP receptor has been demonstrated in the adipose tissue, while GLP-1 receptor does not appear to be relevant [50]. Several studies have shown that GIP plays a role in the metabolism of adipose tissue by increasing the entry of FFA into adipocytes with a consequent reduction in their plasma levels (Figure 2). In animal studies, GIP is able to increase the activity of lipogenesis and lipoprotein lipase. In addition, the possible role of GIP as a pro-inflammatory factor in adipose tissue has also been considered [51].

Stimulation of the FFA receptor GPR120 activates lipogenesis, which helps to maintain lipid homeostasis and reduce insulin resistance. GPR120 is coupled to Gq protein and it induces an increase in intracellular calcium concentration and ERK phosphorylation, both relevant for stimulating adipogenesis [52].

Lipid metabolism in adipocytes is also influenced by β-adrenergic receptors that induce lipolysis by increasing cAMP levels and consequently activating the PKA pathway; on the contrary, insulin counteracts lipolysis and promotes lipogenesis by activating the Akt pathway. However, despite producing this opposite effect, insulin and catecholamines seem to converge in some common denominators, such as Akt activation. Interestingly, insulin resistance and T2D do not alter the hormonal control of lipolysis and the release of FFA [53].

In the white adipose tissue, serotonin, by stimulating 5-HT_2A/2B_ receptors, promotes adipogenesis and absorption of lipids from the bloodstream while simultaneously it inhibits lipolysis induced by β-adrenergic receptors. Whereas in brown adipose tissue, serotonin reduces cellular activity and energy expenditure [47].

Finally, ghrelin causes an increase in the mass of white adipose tissue via a GHS1 receptor-dependent mechanism that activates intracellular mTOR-PPAR signaling pathway, and furthermore, the elimination of GHS receptor reduces the absorption of lipids and lipogenesis [41].

### 3.4. GPCRs and Skeletal Muscle

The GLP-1 receptor seems to have an unusual activity in muscle tissues, such as increasing the synthesis of glycogen and the absorption of glucose in an action similar to insulin [54], and this effect does not involve cAMP but instead the second messenger inositolphosphoglycan (IPG). Conversely, glucagon receptors, expressed at low concentrations in the skeletal muscle might reduce absorption of glucose [55] (Figure 2). The exposure of skeletal muscle to catecholamines induces a slight increase in lipolysis in obese subjects; however, this does not seem to happen in lean subjects, probably due to a different composition of muscle fibers and their sensitivity to β_2_-adrenergic receptors [56]. Both catecholamines and serotonin, through Gs protein-coupled receptors, increase cAMP levels and cause glycogen depletion in rat muscle cells [57].

Histaminergic H_1_ receptors have an important role in regulating glycogen synthesis in muscle tissues. Their stimulation activates some key proteins for intracellular signaling of the insulin receptor, including insulin receptor substrates IRS1/2, and Akt, thereby inhibiting GSK3 and increasing glycogen synthesis. Furthermore, the H_1_ receptors also influence the AMPK pathway, and this could increase the membrane translocation of glucose transporter type 4 (GLUT-4) [58].

On this topic, the 5-HT_2A_ receptor also appears capable of increasing the absorption of glucose in the muscle by inducing greater expression of the transported GLUT-4. In fact, the 5-HT_2A_ antagonists inhibit muscle uptake of glucose [59].

## 4. Effects of AAPs on Hypothalamus and Peripheral Tissues

The AAPs-induced MetS is the consequence of a very complex and broad activity of these drugs on the CNS and peripheral organs, especially by interfering with the activity of GPCRs expressed in these tissues. In addition, if we consider how the CNS through hypothalamus deeply regulates the peripheral organs by altering the concentrations of neuromodulators and hormones in the blood system, the role of the CNS and each organ in the genesis of the AAPs-induced MetS becomes difficult to establish. Therefore, in order to understand the mechanism of MetS, especially in terms of hyperglycemia and dyslipidemia, it is desirable to analyze separately the contribution of the hypothalamus and each relevant organ targeted by AAPs.

### 4.1. AAPs and Hypothalamus

Hypothalamus is the main sensor of the nutrient concentrations in the blood, such as glucose, and it strongly intervenes by influencing glucose and lipid metabolism. Hypothalamus controls glucose and lipid homeostasis coordinating several organs such as the liver, pancreas, adipose tissue, and skeletal muscle, via the autonomic nervous system and the neuroendocrine system. Sympathetic stimulation increases noradrenaline and adrenaline levels, glucagon secretion, production of glucose and lipolysis, and it reduces insulin secretion leading to transitory blood glucose increase. Conversely, parasympathetic activation causes the opposite i.e., increases insulin secretion and inhibits production of glucose [60].

AAPs strongly interfere with hypothalamic centers activity by targeting monoaminergic GPCRs, thus altering descending and ascending autonomic system control. Monoaminergic neurons in the basal brain secrete dopamine, serotonin, noradrenaline and histamine influencing hypothalamic arcuate (ARC) and paraventricular nuclei (PVN) through the GPCRs expressed in these areas.

Among the GPCRs targeted by AAPs, antagonism at H_1_ receptor has been implicated in hyperphagia, weight gain and metabolic dysregulation [61]. Importantly, H_1_ seems relevant in controlling glucose and lipid homeostasis independent of weight gain (Figure 2). A significant association has been found between the genetic variants of H_1_ (rs346074-rs346070) and obesity [62].

A confirmation of the implication of H_1_ in the mechanism of AAPs comes from the observation that AAPs with high affinity for this receptor, such as clozapine and olanzapine, are the strongest for inducing weight gain and metabolic alterations. However, H_1_ antagonism is not the only mechanism involved in the hypothalamus because AAPs have a greater effect on MetS compared to selective antihistaminergic drugs [63]. In fact, dopaminergic and serotonergic antagonism of AAPs have a great impact on specific hypothalamic ARC neurons.

It is known that dopamine is relevant for food intake by regulating α-MSH and orexin expression, therefore D_2_ antagonism can cause an increase of hunger and thus weight gain. The ARC nucleus has a high concentration of D_2_ and D_3_ receptors [64], and their blockade can contribute to the deregulation of glucose and lipid metabolism. In addition, D_2_ antagonism induces hyperprolactinemia with consequences in food regulation and metabolism.

Regarding the role of serotonin in the hypothalamic centers, serotonergic neurons project to the pro-opiomelanocortin (POMC) centers of the ARC nucleus that express 5-HT_2A/2C_ receptors, which reduce the appetite by increasing α-MSH secretion from POMC neurons that leads to melanocortin 4 receptors (MC4Rs) activation [65,66]. ARC nucleus is a relevant sensor for energetic homeostasis because it is sensitive to various neuro-humoral stimuli such as leptin, orexin, insulin, GLP-1, cholecystokinin and ghrelin [63].

Clozapine and olanzapine are potent 5-HT_2A/2C_ antagonists and this is relevant for their effect on glucose and lipid metabolism, and weight gain [67]. In animal models, administration of selective 5-HT_2A/2C_ antagonists resulted in significant increases in insulin resistance and insulin secretion in response to glucose stimulation. Conversely, 5-HT_2C_ agonists have been proposed for the treatment of hyperglycemia and weight gain, and they have been experimented in clinical practice [68].

Alterations in hypothalamic orexigenic and anorexigenic neuropeptides expression and function, including neuropeptide Y (NPY), agouti-related protein (AgRP) and melanocortins have also been demonstrated during AAPs treatment, mainly due to their interference with monoaminergic systems.

In several experiments, it has been shown that AAPs, particularly clozapine and olanzapine, reduce the expression of the anorexigenic peptide POMC and conversely increase the expression of the orexigenic peptide AgRP and NPY [69].

As a consequence of these remarkable changes in the hypothalamus, AAPs increase the sympathetic outflow in the periphery by increasing glucagon secretion [70]. Enhanced levels of norepinephrine have been found in schizophrenic patients taking clozapine and risperidone, but not haloperidol [71]. As a confirmation of implication of the sympathetic system activation, the use of α_2_ or β-adrenergic receptor antagonist with clozapine was able to reverse hyperglycemia and reduce insulin secretion caused by clozapine [72].

Another important target whose function is altered during AAPs use is hypothalamic AMPK. AMPK, highly expressed in the ARC and ventromedial hypothalamus, is a key player in the regulation of energy homeostasis and metabolism, which is influenced by several neurotransmitters and neuropeptides through receptors that are targeted by AAPs [73]. When glucose levels decrease in the brain, AMPK is activated to replenish these levels that are essential for neuronal activity. Consequently, AMPK activates hepatic gluconeogenesis and glycogenolysis via stimulation of the sympathetic nervous system, which increases the secretion of hormones, such as glucagon, corticosterone and epinephrine [74]. AMPK activity is regulated by nutrients, anorexigenic (e.g., leptin) and orexigenic signals, and AAPs hinder these sensing mechanisms.

AAPs are known to increase hypothalamic AMPK activity mostly as antagonists at H_1_ receptors. In fact, AAPs with high affinity for H_1_, such as clozapine and olanzapine, are the strongest AMPK activators, whereas others, such as ziprasidone or lurasidone, have a minimal effect [75]. A study confirmed this hypothesis where clozapine was unable to activate hypothalamic AMPK phosphorylation in H_1_ KO mice, and thus its effect on food intake or weight gain disappeared in these mice [75]. On this line of evidence, the histaminergic stimulant betahistine partially reversed the changes in hypothalamic AMPK activation and NPY expression caused by olanzapine [73].

It should be noted that while AMPK activation in the hypothalamus increases glucose levels and alters lipid metabolism, the same mechanism has an opposite effect in the peripheral organs, such as the liver where it lowers glucose production and increases fatty acid oxidation.

### 4.2. AAPs and Liver

The liver is the principal location of glucose production, and it has a central role in the regulation of systemic glucose and lipid fluxes during feeding and fasting. Since the pancreatic veins drain directly into the portal venous system, every hormone secreted by the pancreas, such as insulin and glucagon, pass through the liver before entering into the systemic circulation. About 70% of hepatic glucose output takes place via glycogenolysis and the remaining 30% via gluconeogenesis [76].

The AAPs-induced increase of glucagon targets the liver that is the principal source of glucose production, and this excessive production is responsible for the development of T2D, even at early stages (Figure 2). In fact, clozapine and olanzapine may induce an increase in glucagon levels even when peripheral glucose levels are high [77]. Glucagon increases the expression of glucose 6-phosphatase (G6Pase) and phosphoenolpyruvate carboxykinase (PEPCK) in the hepatocytes [78], which determine the rate of hepatic glucose production.

A confirmation of AAPs-induced glucagon increase for the hyperglycemic effect is based on early data where olanzapine-induced increase of blood glucose levels was found abolished in glucagon receptor KO mice [79]. Besides glucagon increase, the diabetogenic effect induced by AAPs such as clozapine and olanzapine is also mediated by hepatic insulin resistance as demonstrated in preclinical and clinical studies [80], which contributes to an abnormal increase of hepatic glucose output [77,81]. This effect was however not observed with risperidone [81]. The hepatic insulin resistance induced by clozapine can indeed occur rapidly following a single dose as demonstrated in healthy animals [72].

In relation to hepatic metabolism, AMPK is known to be a key player for glucose homeostasis because its activation lowers blood glucose levels, mostly by inhibiting gluconeogenesis. AAPs significantly decrease AMPK activity thereby altering glucose metabolism [82]. In addition, since activated AMPK also regulates the lipid metabolism by inhibiting lipogenesis and increasing fatty acid oxidation, it is reasonable to assume that AAPs increase hepatic lipogenesis through AMPK inhibition, which contributes to liver fat accumulation [83]. AMPK activation inhibits mTORc1 function, therefore AAPs indirectly stimulate mTOR signaling that increases the expression of the transcriptional activation of sterol-regulatory element-binding proteins SREBP-1c [83]. SREBPs play a central role in controlling a variety of lipid biosynthetic pathways, regulating genes for lipid and cholesterol biosynthesis. AAPs-induced hepatic overexpression of SREBP-1c is relevant for lipid accumulation and liver steatosis. These adverse effects have been demonstrated for clozapine, olanzapine, and risperidone, while aripiprazole or haloperidol did not induce the same effect [84].

SREBPs activity appears to be controlled by the downstream pathways of different receptors present in the liver, and among them, 5-HT_2_ and H_1_ have received particular attention, whose expression is increased in the liver of patients chronically exposed to AAPs [83,85].

In addition, it has been demonstrated that AAPs decrease the transcriptional activity of PPAR, another critical regulator of lipolysis and fatty acid oxidation in the liver but also in the adipose tissue [83].

Pharmacological treatments with antidiabetic drugs such as metformin and PPAR agonists reduce hepatic steatosis and glucose production through activation of AMPK and inhibition of SREBP elements, acting opposite to the mechanism of AAPs.

### 4.3. AAPs and Pancreatic β-Cells

AAPs treatment is associated with T2D, an undesired effect independent of weight gain. Considering that hyperglycemia and peripheral insulin resistance are common side effects induced by AAPs, a compensatory hyperinsulinemia should be expected regardless, and this makes difficult to establish the direct or indirect effects of AAPs on insulin secretion by the pancreatic β-cells. In fact, determining the effect of AAPs on β-cells activity is challenging because conflicting results have been reported. Few in vitro studies demonstrated that clozapine and olanzapine increase basal insulin secretion whereas haloperidol, ziprasidone or aripiprazole did not induce the same effect [86]. In one study, clozapine quickly increased basal insulin secretion at high concentrations; however, it did not alter glucose-stimulated insulin activity [87]. In vitro early studies proposed an inhibitory effect induced by some APs on GSIS [88].

These changes were later confirmed in clinical practice where 30–60% of patients using clozapine or olanzapine showed hyperinsulinemia; however, it is not clear whether this was a compensatory mechanism to insulin resistance or a direct consequence of β-cells stimulation by AAPs [87]. Besides, chronic hyperinsulinemia becomes an aggravating factor for β-cells deterioration and for early onset of T2D.

In relation to the mechanism of AAPs-induced hyperinsulinemia, D_2_, D_3_, 5-HT_2A/2C_ and 5-HT_1A_ receptors expressed in the β-cells might be partially responsible, considering that peripheral dopamine and serotonin generally inhibit insulin secretion in a subtle way (Figure 2). In fact, D_2_ antagonists, such as AAPs and TAPs, slightly increase insulin secretion, while dopamine agonists have an opposite effect [30]. Dopamine seems to be produced in the β-cells starting from its precursors present in the diet and/or from noradrenergic fibers, and in addition, it has a paracrine and autocrine inhibitory function on insulin secretion via D_2_ and D_3_ receptors [30].

Similarly, studies conducted on animal models demonstrated that serotonergic agonists and antagonists have an opposite effects on β-cells insulin secretion by acting on different serotonergic receptors such as 5-HT_2A_, 5-HT_2C_, and 5-HT_1_ [36].

Therefore, it is plausible that the combination of dopaminergic and serotonergic antagonism might contribute to AAPs-induced insulin hypersecretion, more than compounds that are selective at just one receptor subtype. Additionally, the antimuscarinic properties of some AAPs, such as clozapine and olanzapine, have an impact on the parasympathetic control of β-cells insulin secretion. On this aspect, the effect of AAPs is controversial because besides blocking muscarinic receptors, clozapine and olanzapine seem to increase the parasympathetic cholinergic output that probably leads to a rebound on the vagal system with compensatory stimulation of the β-cells [63,89]. In a study, olanzapine administrated for 9 days increased insulin and C peptide secretion that was reduced by atropine treatment, thus confirming the role of muscarinic receptor stimulation [90]. Clozapine and olanzapine-induced insulin dysregulation may also be partly due to blockade of hypothalamic M_3_ receptors [91].

Besides, the antagonism of clozapine at α_2_ receptors expressed on β-cells could reduce the inhibitory effect of the sympathetic system on insulin secretion, and thus have a role in hyperinsulinemia.

To demonstrate the difficulty in establishing definitive evidence on AAPs effect on β-cells insulin secretion, a recent in vitro study found that olanzapine actually reduces insulin secretion in the nanomolar concentration, whereas other works were conducted mostly at higher concentrations. The study proposed that blockade of D_3_, 5-HT_2B_, 5-HT_2C_ and H_1_ receptors expressed in β-cells could be the mechanism responsible for AAPs-reduced insulin secretion [92].

In conclusion, the effect of AAPs on β-cells insulin secretion is still controversial and the hyperinsulinemia caused by AAPs as reported by several papers could be due to peripheral insulin resistance and/or β-cells overstimulation.

### 4.4. AAPs and Adipose Tissue

Data from animal and human studies have confirmed an increase of lipogenesis, visceral fat reserves, FFA circulation and differentiation of pre-adipocytes during AAPs treatment [93]. Studies on cultured cells have shown enhanced lipogenesis in rat adipocytes treated with olanzapine and clozapine [94]. In vivo and in vitro studies both have suggested that clozapine and olanzapine may increase lipogenesis through SREBP1c up-regulation [95], a transcription factor for several genes that are implicated in lipid metabolism (Figure 2).

Undesired changes in adiposity and insulin sensitivity were observed after 12 weeks of AAPs treatment in young patients, including greatest fat increase with olanzapine. In fact, several trials have shown that this phenomenon is more relevant in patients treated with olanzapine and clozapine compared to other AAPs [96,97].

In addition, AAPs differ from other drugs because they are capable of inducing hypertriglyceridemia. Generally, clozapine and olanzapine have the highest propensity to increase triglycerides levels, while quetiapine and risperidone are associated with moderate risk, whereas ziprasidone, lurasidone and aripiprazole seem to have a minimal risk [98].

Besides these effects, AAPs such as olanzapine and clozapine also increase plasma FFAs levels in patients and healthy persons. It is known that increased FFAs impair the ability of insulin to suppress hepatic glucose production and stimulate glucose uptake by skeletal muscle [99].

FFAs are generally sequestered from the plasma by hepatocytes, myocytes and adipocytes and then transformed into activated fatty acids, which are subsequently metabolized via oxidation or conserved via lipogenesis. When FFAs production exceeds the capacity of these two pathways to dispose them, then FFAs and their intermediates have a negative effect on insulin action at the cellular level. Some studies have illustrated that an excess of FFAs induces insulin receptor inactivation and degradation, including disruption of IRS1 [100,101].

Besides metabolic alterations, it has been demonstrated that AAPs are able to stimulate the differentiation of preadipocytes into adipocytes, thereby contributing to adipose tissue mass. In fact, olanzapine and clozapine were found to upregulate the expression of transcription factors essential for adipocyte differentiation [102]. Due to increased adiposity, a rise in leptin levels generally occurs, which in the long term can lead to leptin-resistance in the hypothalamic centers, thereby altering appetite regulation [103]. Recently, it was shown in 140 subjects that low adiponectin and high leptin levels in dysfunctional adipose tissues may contribute to increased oxidative stress and inflammation, which can be a marker for severity of MS [104].

All these above-mentioned alterations indicate the relevance of adipose tissue in the onset of MetS that causes insulin resistance, overstimulation of β-cells insulin secretion and inflammation, which in the long-term could lead to T2D and obesity.

### 4.5. AAPs and Skeletal Muscle

Based on the evidence that the H_1_ and 5-HT_2A_ receptors play an important role in glycogen synthesis and glucose uptake mostly through GLUT-4 in muscle cells, it is reasonable to expect that glucose metabolism might be locally impacted by AAPs having strong antagonism at H_1_ and 5-HT_2A_, especially by reducing plasma glucose clearance that contributes to the onset of T2D [59] (Figure 2).

In an in vitro study, olanzapine inhibited IRS-1 and Akt phosphorylation induced by insulin and impaired insulin-signaling cascade, and this can be relevant for the peripheral insulin resistance [105]. Similar evidence was also found for clozapine in L6 muscle cells; however, ex vivo studies on rat-isolated skeletal muscle found contradictive results for AAPs [59].

Another factor that reduces skeletal muscle sensitivity to insulin is the increase of plasma FFA. As aforesaid for the adipose tissue, FFA can also interfere negatively with insulin receptor downstream signaling in the muscle cell and induce receptor degradation as well [101].

In addition, lipidomic studies on lipid contents (e.g., FFA, phosphatidylcholines and ceramides) in skeletal muscle biopsies from patients treated with AAPs have found several differences compared to the general population with potential consequences in insulin resistance; conversely, mood stabilizers did not cause the same shift [106].

## 5. AAPs and MetS in Clinical Practice

AAPs are generally considered well-tolerated especially in terms of motor side effects; however, they often cause MetS, which is responsible for reduced life expectancy and poor adherence to the therapy. Importantly, there is clear evidence that early metabolic changes can precede weight gain, an aspect that needs to be properly monitored [9].

Several reviews and meta-analysis that systematically compared AAPs with TAPs in terms of efficacy and side effects found data supporting AAPs superiority for several aspects, but with an increased risk of developing MetS [8,107].

However, in terms of MetS, AAPs are a heterogeneous class of drugs. In fact, olanzapine and clozapine are associated with the greatest risk, whereas quetiapine, risperidone, asenapine, and amisulpride show a moderate level of this undesired effect (Table 1). Interestingly, ziprasidone and lurasidone seem more tolerable on the metabolic profile; however, these drugs are relatively new thus their therapeutic efficacy in comparison with other AAPs still needs to be determined. A reduced level of MetS has also been found for aripiprazole, an AAP with a different receptors profile compared to the other drugs [8,98]. The prevalence of clozapine and olanzapine-induced MetS is the highest compared to the other AAPs, and it has been reported to be between 25% to 50% [108,109]. In addition, AAPs polypharmacy is generally associated with the increasing risk of MetS [110].

TAPs have a lower tendency to produce MetS compared to AAPs, but they might still induce it, indicating that the D_2_ receptor blockade has consequences on the metabolism. Some authors suggested that haloperidol might lead to weight gain and metabolic disturbances due to its activity on hypothalamic centers and also to hyperprolactinemia [111]. Surprisingly, the METEOR study (Evaluation of Metabolic disorders in Schizophrenic patients) in 2006, conducted an analysis to evaluate the differences between TAPs and AAPs for inducing MetS and it found more similarities than expected, in contrast with many other meta-analyses, such as the CATIE (Clinical Antipsychotic Trials of Intervention Effectiveness) study, where the difference was clear. Therefore, even if this study can be downsized, it still warns about the risk of metabolic alterations associated to the use of TAPs [95].

In relation to weight gain, several meta-analysis studies have consistently shown that clozapine and olanzapine are associated with the highest risk, followed in order by quetiapine, risperidone, paliperidone, and asenapine, whereas the other AAPs, such as ziprasidone, lurasidone and aripiprazole seem less detrimental on this effect [107].

In the first CATIE study, olanzapine showed a weight gain of almost 1 kg/month, and a similar conclusion was reached in a following trial over one-year in which olanzapine and clozapine induced a weight gain of approximately 12 kg while quetiapine and risperidone increased weight of 2–3 kg [112]. Aripiprazole and asenapine showed relatively little weight gain where apparently ziprasidone did not show any gain [113,114].

In another study, Fountaine et al. (2010) showed in healthy volunteers how olanzapine treatment increased the calories intake of 345 kcal per day and the resulting weight gain of 2.6 kg after 2 weeks. A similar pattern was found in olanzapine-treated schizophrenic adolescents for 4 weeks, confirming the AAP-induced increase of appetite and excess caloric intake coupled with increased water retention [115].

Besides causing weight gain, another undesired effect caused by AAPs treatment is fasting hyperglycemia, which can develop in T2D. Most importantly, this side effect is not strictly dependent on weight gain, and 25% of the cases of T2D involve people that are not obese [116]. In fact, the development of hyperglycemia and insulin resistance can happen in the early period of AAPs treatments (within 6 months) and precede weight gain. Besides, in the long-term, weight gain undoubtedly becomes an aggravating factor for T2D.

A meta-analysis on 270,000 subjects found that clozapine and olanzapine are associated with an increased risk of T2D, while risperidone or quetiapine show a moderate risk. T2D prevalence with the use of AAPs has been shown to range from 3% to 28% [117].

Seven out of nine studies showed a significant association between olanzapine and T2D, whereas three out of four studies found the same evidence for clozapine [118,119]. Risperidone (five out of nine) and quetiapine (three out of six) also showed a positive association with T2D, but to a less degree. Evidence for aripiprazole was discordant probably indicating a lack of clear association [119]. Other AAPs such as ziprasidone, lurasidone, and asenapine are associated with low risk of T2D. Another study indicated that the risk of olanzapine-induced T2D was dose-related, while quetiapine and risperidone showed increased risk only at high doses [120]. Although T2D seems rare in AAP-treated adolescences, there is evidence of a potential risk based on the duration of the treatment and on the use of polytherapy [121].

When obesity develops during AAPs use, insulin sensitive tissues become more resistant to insulin and β-cells increase insulin secretion in order to compensate, which in the long term can determine β-cells deterioration thus increasing the risk of T2D.

AAPs treatment is also associated with dyslipidemia with rates between 15–50%. Saari et al. studied a cohort of 5654 patients in Northern Finland and found that the risk of dyslipidemia in individuals treated with AAPs was increased by 2.8 times for hypercholesterolemia, 2.3 times for hypertriglyceridemia and 1.6 times for high LDL cholesterol, and this increase was associated with weight gain [122]. A systematic review comparing metabolic effects in different AAPs found that olanzapine and clozapine have the highest risks for increased cholesterol in addition to weight gain [119]. Another study found that olanzapine caused the highest cholesterol elevation and increased waist circumference among different AAPs [109]. Despite the relevance of dyslipidemia and hyperglycemia in schizophrenic patients, most of them are not treated pharmacologically. For example, Mackin et al. found that only 7% of patients were receiving lipid-lowering therapy [123]. In addition, a high percentage of schizophrenic patients had cardiovascular problems [124].

When any of the above-mentioned metabolic abnormality occurs in patients, evidence from clinical trials suggests that switching AAP medication from one with high risk of MetS to another with lesser risk potential could be a potential way to manage these side effects, but the data are still limited so further examinations are required [125].

Though we focused our analysis on the most used AAPs, one should note that the more recent ones, such as cariprazine, brexpiprazole, and lumateperone [126], have shown a lower impact on metabolic parameters (BMI, weight change, LDL levels) when compared to clozapine or olanzapine [98]. Regarding the mechanism of action of these new AAPs, we suggest to refer recent reviews by Torrisi et al. and Caraci et al. [127,128].

## 6. Treatments for AAPs-Induced MetS: Pharmacological and Non-Pharmacological Interventions

### 6.1. Non-Pharmacological Interventions

The importance of an intervention aimed at decreasing the risk of AAPs-induced MetS is crucial considering how it can have a significant impact on patient’s health conditions and compliance, especially in the long term. In fact, sedentary lifestyle and high-caloric diet, in association with the use of AAPs, can be detrimental for weight gain, T2D and dyslipidemia. In addition, weight gain increases the social stigma associated with mental illness, and it often pushes the patients to abandon their pharmacological therapy.

The first-line treatment for AAPs-induced MetS is based on psychoeducation, physical exercise and diet. Many studies have underlined how different strategies based on psychoeducation, psychotherapy and health programs can succeed in preventing and/or reducing the onset of AAPs-induced MetS [129].

A meta-analysis of 17 trials related to psychoeducational, cognitive-behavioral, nutritional and physical activity interventions collected evidence on the effectiveness of health promotion programs for weight loss and prevention of weight gain in psychotic patients. The analysis found a substantial and relevant decrease of more than 3% of the initial weight; besides, the guidelines of the Occupational Medical Service (OMS) and National Institute of Health (NIH) indicate a weight loss of 5–10% as the optimal criterion for success [130]. On this topic, as a “lifestyle therapy,” the NIH has provided a practical guidance based on correct diet, increased physical activity and behavioral therapy. The behavioral strategies are focused on improvement of the executive functions, problem-solving skills and anti-stress techniques to be carried out for at least 6 months.

Scientific literature shows various intervention programs starting from simple commercial manuals to structured psychological therapies, differing in terms of modality, duration and number of sessions [129].

For example, a study based on the Weight Watchers program, which combines a regulated diet and motivational psychology, demonstrated weight reduction by 2.3 kg in patients taking olanzapine compared to 0.2 kg in control group [131].

Furthermore, Barton et al. have pointed out that each therapy should be tailored to patient-specific characteristics, particularly considering the severity of the symptoms, and adopt an approach that encourages small and feasible daily changes such as eliminating sugary drinks and adding 2000 steps each day [129,132]. Similar evidence emerges from a case-controlled study conducted on 70 patients treated with olanzapine, where a healthy intervention program seems to be effective in maintaining body weight up to 4 months [133]. Other educational strategies that were successful in preventing or reducing weight gain include a multidisciplinary approach based on strong motivational support (e.g., to have a diary to record food intake and exercise) and changing incorrect food habits [134,135].

In addition, besides weight control, psychoeducation can be important for trying to make patients aware of their conditions and on the side effects of the pharmacological treatments to help improve patient’s adherence. On this topic, there are also even more structured treatments such as cognitive behavioral therapy (CBT) techniques. Weber et al. (2006) examined the positive results achieved through a group cognitive-behavioral intervention (1 h per week for sixteen weeks) in a diabetes prevention project on patients taking AAPs [136].

Most psychological interventions focus on change of dysfunctional beliefs and cognitive bias towards unhealthy food habits, and on improvement of emotional states and sensations related to hunger and satiety, thus promoting healthy behaviors in relation to food. In addition, other approaches such as role-playing, problem-solving exercises, motivational encouragement, and the use of diary to record food activities and exercise, all might contribute to control weight gain and MetS.

### 6.2. Pharmacological Interventions

When diet or any non-pharmacological intervention tried as first-line treatment is unable to reduce the AAP-induced MetS, it often becomes necessary to introduce an appropriate pharmacological therapy. It is quite surprising to notice that a large numbers of patients with psychotic disorders having MetS are not pharmacologically treated with AAPs [137].

In this direction, metformin appears to be one of the most effective drugs to counter weight gain and T2D induced by AAPs (Table 2). Metformin belongs to the biguanide family, and it is the main drug used for treating T2D and MetS. By activating AMPK in the liver and other tissues, metformin reduces hepatic glucose production and lipogenesis, stimulates fatty acid oxidation, and facilitates glucose uptake from the blood [138]. Jesus et al. have showed that metformin reduces weight gain and improves insulin sensitivity as well [139]. In a recent review, metformin was shown to be effective on weight gain and insulin resistance in four out of five studies performed in patients taking AAPs with metabolic adverse reactions [95].

A study on adult rats treated with olanzapine tested the efficacy of different class of antidiabetic drugs in combination. Co-administration of metformin with glibenclamide, or with rosiglitazone or with glibenclamide, reduced glucose levels more than the single treatments, efficiently counteracting the hyperglycemic effect of olanzapine [150].

The addictive effects of the two drugs can be explained by their complementary action, since glibenclamide acts on the pancreatic β-cells whereas metformin and rosiglitazone regulate mostly the metabolism in the liver and in the adipose tissues, respectively [91]. According to these authors, the increase in insulin levels alone is not sufficient to cause a reduction in hyperglycemia caused by AAPs, as the involvement of other mechanisms seem relevant. In addition, metformin seems to counter some of the AAPs effects in the hypothalamic centers. In fact, when prolonged treatment with olanzapine results in an increase in hypothalamic phosphorylation of AMPK, metformin reduces AMPK activity with consequences in the food intake and glucose homeostasis [151].

Intriguingly, oral administration of metformin also increased postprandial GLP-1 levels in diabetic patients, which seems to contribute to metformin’s glucose-lowering effect through AMPK-dependent effect on GLP-1–secreting intestine L cells. In addition, metformin seems to have cytoprotective properties by stimulating proliferation of pancreatic β-cells and reducing cellular apoptosis [152]. In 2006, GLP-1 analogues (lixisenatide, liraglutide and dulaglutide) were approved for T2D and, at higher doses, they also reduce weight [153].

Lykkegaard et al. (2008) conducted an experiment on female rats treated with olanzapine for 14 days followed by GLP-1 analogue liraglutide. Liraglutide succeeded in reversing the weight gain caused by olanzapine, reduced food intake, normalized glucose tolerance and demolished mesenteric and retroperitoneal fat stores in rats [154]. A case report of a schizophrenic woman with T2D triggered by clozapine when treated for 3 months with liraglutide demonstrated a loss of weight of 6 kg and the glycated hemoglobin value was stabilized at 6.1% [155].

Considering that AAPs increase hunger, a potential pharmacological strategy is to try drugs that decrease appetite. One option is reboxetine, a selective norepinephrine reuptake inhibitor. The increase of norepinephrine can be effective in weight reduction by reducing appetite [156]. A double-blind placebo-controlled study evaluated the ability of reboxetine to prevent or mitigate weight gain caused by olanzapine. Results showed that the control group treated with olanzapine and placebo gained an average of 5.5 kg in 6 weeks, while the group treated with olanzapine and reboxetine only gained an average of 2.5 kg [157].

On this topic, the use of the selective serotonin reuptake inhibitor (SSRI) fluoxetine is controversial, as clear evidence was not demonstrated, besides small improvements that appeared were transient [158].

On olanzapine-induced weight gain, amantadine was found to be effective in losing an average of 3.5 kg in 6 months on a small number of patients [159], an evidence which was later confirmed in a larger study [160].

Topiramate is another drug that has been studied for reducing weight gain where weight loss has been reported in 10–20% of patients [161], which depended on several factors such as initial body-mass index (BMI) and duration of the treatment [162]. The anorexigenic capability of topiramate appears to be linked to the reduction of glutamatergic activity [163] and to changes in the neuropeptide Y expression in the hypothalamus [164].

Another therapeutic strategy aimed at controlling weight gain is histamine H_2_ receptor antagonism. Nizatidine, an H_2_ antagonist used primarily as an anti-ulcer drug, was able to lower olanzapine-induced body weight and leptin serum levels [165,166].

For AAP-induced dyslipidemia, one pharmacological strategy is the use of statins, such as simvastatin, lovastatin, atorvastatin and rosuvastatin, as several studies support their use in schizophrenic patients [1,157,158]. In a recent in vivo study by Liu et al. (2019), administration of simvastatin concomitantly with olanzapine reduced plasma lipid levels and liver fat concentrations in rats. Simvastatin resulted in down-regulation of SREBP-1, FASN (fatty acid synthase), CLY (ATP citrate lyase), ACC (acetyl-CoA-carboxylase), and SCD-1 (stearoyl-coa-desaturase-1) genes, whose expression is increased after olanzapine treatment. Furthermore, the hepatic expression of mTORc1 decreased after treating with statins. In the liver, mTORc1 is associated with lipid biosynthesis [167] and it can be overactivated by AAPs [168]. Statins also reduces many inflammatory indices such as C-reactive protein, interleukin-1β, and tumor necrosis factor-α [169].

Another drug used for weight control is orlistat, a lipase inhibitor, which should be combined with a reduced fat diet. The advantage of orlistat is that it does not affect the CNS, but it can however affect the gastrointestinal system causing diarrhea, bloating, and cramps [160].

Nevertheless, orlistat seems more effective in preventing weight regain than in promoting weight loss [170].

## 7. Towards a Personalized Therapy: AAPs Plasma Concentration Monitoring and Pharmacogenetics

Despite APs treatments, more than 1/3 of patients do not benefit from the pharmacological therapy. Among the factors responsible for the inadequacy of the pharmacologic treatments, besides using standard dose, suboptimal plasma drug concentration (Cp) is one of them. In addition, unexpected high Cp can be responsible for severe side effects. Both these conditions can have a negative impact on patient adherence and compliance to the treatment. The pharmacokinetic unique characteristics of each individual, especially in terms of metabolism, and/or drug-drug interactions can be responsible for the unexpected results mentioned above [171].

For these issues, TDM is an effective tool to improve adherence to pharmacological treatments in clinical practice by measuring Cp, towards a personalized medicine by identifying each individual patient’s best therapeutic concentration [172]. For AAPs and TAPs, the use of TDM is strongly recommended (Level I recommendation) based on the evidence of a good correlation between Cp, clinical response and the onset of APs-induced severe side effects [171,172].

Neuroimaging studies have demonstrated that motor-related side effects may occur when more than 80% of D_2_ receptors in the striatum are blocked by APs. Importantly, a correlation was found between the D_2_ receptor occupancy and the Cp of some APs, while such a relationship with dose was less clear [173]. One reason for such a discrepancy is related to the APs metabolism that involves different cytochromes such as CYP1A2, CYP2D6, and CYP3A4 that are known to be genetically polymorphic among the general population.

These analyses have also found that the relationship between Cp and D_2_ receptor occupancy is fit by a hyperbolic saturation curve, where risperidone and olanzapine, at higher concentration, may exceed 80% of receptor occupancy [174]. On the contrary, even at high concentrations clozapine never reaches this level of D_2_ receptor occupancy. These saturation curves allow predicting D_2_ receptor occupancy by measuring Cp, a correlation that is especially valid for olanzapine. For risperidone, the analysis becomes more complex because the efflux transporter P-glycoprotein (P-gp) may be responsible for lowering its concentration in the brain, thereby making the relationship between Cp and D_2_ receptor occupancy less predictable. Therefore, possible correlation between Cp and other targets are also being analyzed, such as the 5-HT_2A_ and GlyT1 transporters; however, the results are still preliminary [175].

With reference to clozapine, many TDM studies have been carried out mainly due to its Cp, large variability at standard dose and relevant side effects. Sex, age, smoking and concomitant use of other medications, such as antiepileptics (e.g., carbamazepine) and SSRI (e.g., fluvoxamine), may influence clozapine Cp up to ten times. Similar interactions were also found for other AAPs such as olanzapine and risperidone in association with carbamazepine or SSRIs [176].

A correlation was found between Cp of clozapine and increased risk of epileptic seizures; hence the strong suggestion to use TDM during its usage [177]. Moreover, a fluctuation of Cp during AAPs usage can have a negative effect on relapses and rehospitalization in psychotic patients, where TDM might help reduce such a risk. Finally, higher Cp of AAPs can be associated with an increased risk of metabolic alterations in schizophrenic patients [178].

Taken together, these data clearly show the utility of TDM in patients undergoing AAP treatment. However, in order to obtain relevant benefits, TDM should be properly integrated in the daily clinical practice where a multidisciplinary approach is applied to take advantage of the expertise of each specialist, i.e., pharmacologists and clinician doctors.

When moving towards a tailored therapy, pharmacogenetics could be relevant for characterizing predictive genetic variants affecting Cp, drug response, and adverse reactions. 

Isoforms of CYP enzymes are known to influence the pharmacokinetics of many drugs, and single nucleotide polymorphisms (SNPs) have been reported for AAPs on CYP2D6, CYP1A2, CYP3A4, CYP2C9, and CYP2C19 that are responsible for interindividual variability in Cp, and to some extent drug efficacy. For instance, the CYP2D6 poor metabolizers showed higher plasma levels of haloperidol, aripiprazole, risperidone and other commonly used APs [179]. The genetic variability of CYP1A2, the main metabolic enzyme for clozapine and olanzapine, partially explains the variability of their plasma levels [180]. With reference to efficacy, given the importance of dopaminergic and serotoninergic systems, polymorphisms on the DRD2, DRD3, 5HTR1A, and 5HTR2A receptor genes showed some influence in AAPs response, although the association was modest [179,181]. A recent review and meta-analysis on genetic polymorphisms affecting clozapine efficacy [182] reported only three polymorphisms in serotonin receptor genes related to clozapine efficacy, rs6313 and rs6314 within the HTR2A gene, rs1062613 within the HT3A gene, but no link with induced weight gain. Serotoninergic genes are also involved in metabolic adverse reactions, where polymorphisms on 5HTR2A and 5HTR2C were associated to weight gain, greater waist circumference, increase in BMI and MetS [10,183]. Besides preliminary data, we believe that currently there is no clear correlation between genetic polymorphisms and the risk of MetS during AAP use, demonstrating how several factors (genetic and not genetic) are probably involved for determining this complex phenotype.

## 8. Conclusions

The use of AAPs in clinical practice as first choice for treating psychotic disorders is based on their higher efficacy on the cognitive symptoms and reduced motor side effects compared to TAPs. However, the frequent occurrence of MetS, especially in the terms of T2D and obesity, with AAPs has seriously questioned their overall superiority over the other drugs.

It is surprising how these relevant side effects have not been properly considered in the past, but now finally clinicians are aware of their importance and they are firmly addressing these issues. The need of a prompt intervention to prevent or reduce AAPs-induced MetS is mandatory, and treatments based on psychoeducation, physical exercise and diet have proven to be successful through programs that are feasible and affordable. When these schemes fail, fortunately, there are several pharmacological options available to treat MetS, which have been underused in schizophrenic patients so far.

One should know that the presence of T2D or obesity could negatively affect patient compliance and lead to drug suspension, resulting in relapse and poor clinical outcomes. In addition, weight gain increases the social stigma associated with mental illness, and it often pushes the patients to abandon their treatments.

Olanzapine and clozapine are associated with the greatest risk of MetS; however, these aspects must be considered together with their higher efficacy, where clozapine still remains the most effective AAP. The newer AAPs such as ziprasidone, lurasidone are more tolerable on the metabolic profile, but their overall clinical efficacy is less compared with clozapine.

We might conclude that there is a correlation between AAP’s clinical efficacy and likelihood of metabolic alterations, whereas drugs with the highest activity in terms of number of receptor targets (e.g., clozapine) are the ones with the major risk of MetS.

Indeed, the AAPs-induced MetS is the consequence of a broad activity on the CNS (mostly the hypothalamus) and peripheral organs, especially by interfering with the activity of various GPCRs expressed in these tissues. Finally, it is imperative to have a better understanding of the molecular mechanisms responsible of the clinical differences among the AAPs in order to find new and better drugs.

## Figures and Tables

**Table 1:** Clinical differences between AAPs in terms of negative/cognitive symptoms improvement, MetS (gain, T2D, dyslipidemia) and parkinsonism. Based on reviews and meta-analysis studies [8,107], values are reported as very high (++++), high (+++), moderate (++), low (+), very rare (+/−), and with no effect (0).

AAPs	Negative/Cognitive Symptoms Improvement	Weight Gain	Diabetes (T2D)	Dyslipidemia	Parkinsonism
Clozapine	++++	+++	+++	+++	0
Olanzapine	+++	+++	+++	+++	+/−
Quetiapine	++	++	++	++	+/−
Risperidone	++	++	++	++	++
Amisulpride	++	++	++	++	++
Asenapine	++	+	+	+	+
Lurasidone	++	+	+	+	+
Ziprasidone	++	+	+	+	+
Aripiprazole	++	+	+	+	+

**Table 2:** Clinical studies evaluating pharmacological interventions against AAPs-induced MetS.

Added Treatment	AAP	Study	Pharmacological Response	Reference
**Metformin**	**Olanzapine**	Double-blind study: 25 patients randomly assigned to olanzapine plus metformin or olanzapine plus placebo for 24 weeks.	Metformin-group gained 3% of body weight compared to 7% for placebo group. BMI change was 0.85 in metformin-group vs. 2.02 in placebo-group.	[140]
		40 patients randomly assigned treatment with olanzapine plus metformin or olanzapine plus placebo for 12 weeks.	Metformin-group vs. placebo group resulted in lower increase in body weight (1.90 vs. 6.87), fasting insulin level (0.81 vs. 6.78) and insulin resistance index (0.22 vs. 1.49).	[141]
		80 patients taking olanzapine were randomized metformin or placebo comedication treatment for 12 weeks.	Body weight change was −1.4 in metformin-group and non-significant in placebo. Insulin resistance increased after placebo and not after metformin.	[142]
		40 patients taking olanzapine were assigned to metformin or placebo for 14 weeks.	No significant improvements for treated vs. placebo group.	[143]
	**Clozapine**	55 subjects taking clozapine for at least 3 months, were assigned to metformin or placebo for 24 weeks.	Body weight, BMI, fasting plasma glucose, HDL, insulin level had significant changes in the metformin-group.	[144]
		61 patients treated with clozapine were randomly assigned to metformin extended release or placebo for 14 weeks.	Mean change in body weight was −1.87 kg for metformin-group and 0.16kg for placebo-group Insulin and the triglyceride/HDL ratio significantly decreased after metformin.	[145]
**Rosiglitazione**	**Olanzapine**	12-week double blind study on 30 patients treated with olanzapine were allocated to rosiglitazione or placebo.	Insulin and the insulin resistance significantly decreased after rosiglitazone, while no effect was seen on weight gain and lipid profile.	[146]
	**Clozapine**	8-week double blind, placebo-controlled trial of rosiglitazone 4 mg/day in 18 clozapine-treated schizophrenia subjects with insulin resistance.	Non-significant improvement on glucose utilization and insulin sensitivity index; significant reduction in LDL level in rosiglitazone group.	[147]
**Liraglutide**	**Olanzapine or clozapine**	103 patients with a BMI > 27 and prediabetes randomly assigned to liraglutide or placebo for 16 weeks.	Liraglutide-group (63.8%) developed normal glucose tolerance compared with placebo-group (16%). Liraglutide induced a placebo-subtracted body weight loss of 5.3 kg.	[148]
**Exenatide**	**Clozapine**	28 patients treated with clozapine randomly assigned to exenatide extended release or standard care for 24 weeks.	6 people on exenatide achieved >5% weight loss vs. 1 usual care. Participants on exenatide had greater weight loss (−5.29 kg vs. −1.12 kg), BMI reduction (−1.78 vs. −0.39), reduced fasting glucose (−0.34 vs. 0.39) and HbA1c (−0.21 vs. 0.03) compared to control.	[149]
